# Prognostic value of the bone scan index in patients with metastatic castration-resistant prostate cancer: a systematic review and meta-analysis

**DOI:** 10.1186/s12885-020-06739-y

**Published:** 2020-03-20

**Authors:** Hualin Song, Song Jin, Peng Xiang, Shuai Hu, Jie Jin

**Affiliations:** 1 Department of Urology, Peking University First Hospital and Institute of Urology, Peking University, Beijing, 100034 China; 2 National Research Center for Genitourinary Oncology, Beijing, China; 3 Beijing Key Laboratory of Urogenital Diseases (male), Molecular Diagnosis and Treatment Center, Beijing, China; 4 Department of Urology, Beijing Tsinghua Changgung Hospital, Tsinghua University, Beijing, China; 5 Department of Urology, Beijing Tongren Hospital, Capital Medical University, Beijing, China

**Keywords:** BSI, Bone scan index, Metastatic castration-resistant prostate cancer, mCRPC, Meta-analysis

## Abstract

**Background:**

Many studies have reported the prognostic significance of the bone scan index (BSI) for metastatic castration-resistant prostate cancer (mCRPC); however, these reports are controversial. This study investigated the BSI in mCRPC and its relationship with prognosis.

**Methods:**

The PubMed, Cochrane, and Embase databases were searched systematically for relevant articles published before September 1, 2019. Hazard ratios (HRs) were used to investigate the prognostic value.

**Results:**

This study finally identified 9 eligible studies. The results suggested that high baseline BSI predicted poor OS (HR = 1.331, 95% CI: 1.081–1.640) and that elevated ΔBSI also predicted poor OS (HR = 1.220, 95% CI: 1.015–1.467). The subgroup analysis stratified by ethnicity showed that the baseline BSI and ΔBSI predicted poor OS in the Asian population but not in the Caucasian population. We also performed a subgroup analysis based on the different cut-off values of baseline BSI. The subgroup of ≤1 showed a significant association with OS in mCRPC patients.

**Conclusion:**

Our study demonstrated that high baseline BSI and elevated ΔBSI predicted poor OS in patients with mCRPC. Hence, the BSI can serve as a prognostic indicator for mCRPC patients and may therefore guide clinical treatment in the future.

## Background

The early diagnosis of prostate cancer (PCa) has increased since the introduction of the prostate-specific antigen (PSA) blood test > 25 yr ago, but many patients still fail initial treatment and progress to castration-resistant prostate cancer (CRPC) or metastatic castration-resistant prostate cancer (mCRPC) [1–3]. New bone metastases usually occur in CRPC patients, which indicates a high risk of poor outcome [4]. After the development of mCRPC, patients commonly initiate secondary hormonal manipulation or chemotherapy. Sipuleucel-T, abiraterone, enzalutamide, docetaxel, cabazitaxel, and radium-223 have all improved survival among men with mCRPC [4, 5]. However, there are no precise indicators that can predict the prognosis of patients with mCRPC with sufficient accuracy. Many physicians use PSA when following PCa patients with bone metastasis. However, PSA is not a good surrogate marker for mCRPC and can only be used to evaluate the effectiveness of treatment [6, 7]. Therefore, we need new and effective indicators to predict the prognosis of patients with mCRPC.

Bone scintigraphy (BS) is a widely used method to assess metastatic spread within the skeleton, but previously, there was a lack of standardization in its analysis. The bone scan index (BSI) is a kind of bone scan interpretation that estimates the quantitative bone metastasis burden [8, 9], which was originally calculated by individual bone scan readings. The BSI was originally reported in 1998 as an imaging biomarker for bone metastatic prostate cancer [8]. Later, an automated BSI was developed with the use of computer-assisted diagnosis software, making the assessment of metastatic spread more objective and comparable [9–11]. The BSIs subsequently included and analysed in this study are all automated BSIs. Many studies have recently shown that BSI progression or a change in BSI (ΔBSI) during treatment was strongly associated with worse OS in men with mCRPC [9, 12, 13]. Due to differences in study design, sample size, and other factors, the research on the BSI in mCRPC patients has reported some conflicting results. Therefore, it is time to perform a systematic meta-analysis to understand the prognostic value of BSI in patients with mCRPC.

In this study, we evaluated the prognostic role of the baseline BSI and BSI changes in terms of overall survival (OS) in patients with mCRPC by pooling the available outcome data.

## Methods

### Search strategy

We conducted this meta-analysis using a well-recognized protocol based on the Preferred Reporting Items for Systematic Reviews and Meta-Analyses (PRISMA) [14]. The PubMed, Cochrane, and Embase databases were searched systematically for relevant articles published before September 1, 2019. We searched for keywords as follows: “castration-resistant prostate cancer” or “metastatic castration-resistant prostate cancer” or “CRPC” or “mCRPC” and “bone scan index” or “BSI” and “prognosis” or “survival” or “outcome”. All of the included documents were published in English.

### Inclusion and exclusion criteria

All included articles met the following criteria: 1) the baseline BSI and ΔBSI were used to predict OS; 2) all patients were diagnosed with mCRPC; and 3) hazard ratios (HRs) and 95% confidence intervals (CIs) could be obtained from the article.

The exclusion criteria were as follows: 1) articles published in languages other than English; 2) animal studies; 3) studies with incomplete data; and 4) duplicate publications.

### Data extraction

The data were independently evaluated by two reviewers, and if there were inconsistencies, the reviewers discussed them together with the participation of a third author. We assessed the quality of selected items on the basis of the Newcastle-Ottawa Scale (NOS) [15]. A high-quality study was indicated by a score of six or higher. The following information was recorded for each study: first author, year of publication, country of origin, number of patients, cut-off value, HR for survival (OS), and follow-up time.

### Statistical analysis

The statistical analysis was conducted with Stata SE14.0 (Stata Corp LP, USA). HRs and 95% CIs were applied to evaluate the relationships between baseline BSI and OS and between ΔBSI and OS. We used the chi-square test and *I*^*2*^ statistic (100% × [(Q-*df*)/Q]) to evaluate inter-study heterogeneity [14, 15], and a value of P (heterogeneity) < 0.05 or *I*^*2*^ > 50% was considered statistically significant. When the value of P (heterogeneity) is > 0.05 or *I*^*2*^ is < 50%, we choose to use the fixed effects model; otherwise, we choose to use the random effects model. Subgroup analysis were based on ethnicity, divided into Asian and Caucasian populations, and cut-off values for baseline BSI, divided into the ≤1 population and the > 1 population. Sensitivity analysis was performed to evaluate the stability of the baseline BSI and ΔBSI results for OS. The cut-off of the baseline BSI in this analysis was selected based on the cut-off used in the literature included in the study. We chose to use a funnel chart to measure publication bias. *P* < 0.05 indicates statistical significance.

## Results

### Study characteristics

**Fig. 1:**
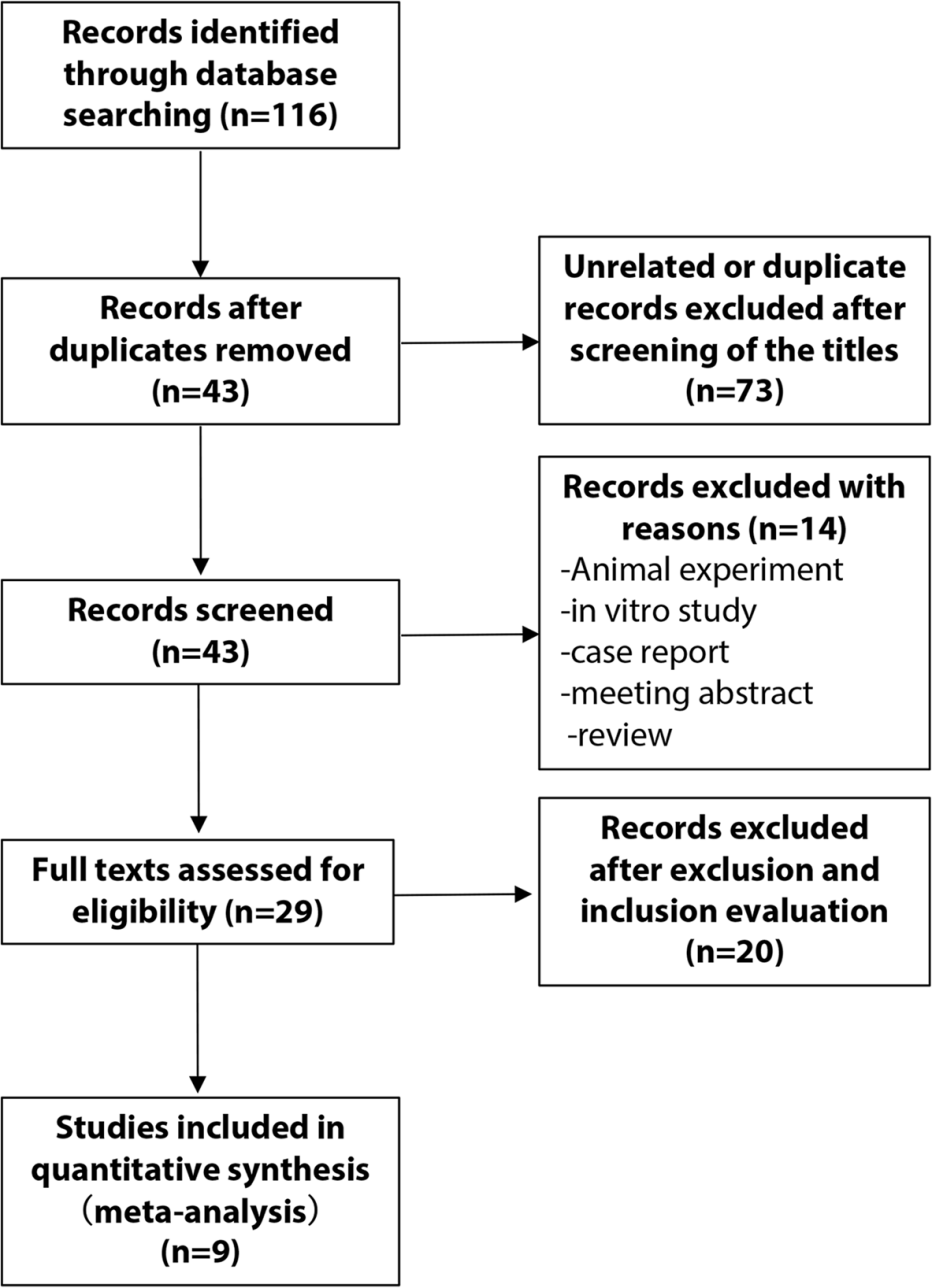
Flow diagram of the study selection process

The search strategy of the current meta-analysis identified a total of 116 studies. Overall, 87 records, identified as irrelevant by title and abstract screening, were excluded, and the full text articles of the remaining 29 records, which investigated the relationship between BSI and survival outcomes of mCRPC patients, were evaluated. According to our inclusion and exclusion criteria, 9 studies [16–24] were eligible and eventually included in our meta-analysis. The flowchart of our study is shown in Fig. 1. The major characteristics of these studies are summarized in Table 1. The number of participants in each study ranged from 31 to 144, for a total of 567 patients. The cut-off value to distinguish high BSI from low BSI was set from 1 to 5 (Table 1). The median follow-up periods ranged from 4 to 40 months.

**Table 1:** Main characteristics of included studies

Study	Year	Ethnicity	Model	No. of patients	Median age	Median follow-up	Cut-off %	Analysis	NOS
Yozo Mitsui	2012	Asian	multivariate	42	73	40	3	Baseline BSI, ΔBSI	7
Andrew J. Armstrong	2014	Caucasian	multivariate	85	–	24	1	Baseline BSI, ΔBSI	6
Yasuhide Miyoshi	2016	Asian	multivariate	40	75.5	–	1	Baseline BSI	7
Koichi Uemura	2016	Asian	multivariate	41	73	17.7	1	Baseline BSI	7
Mariana Reza	2016	Caucasian	multivariate	104	72	13	–	ΔBSI	6
Ajjai Alva	2017	Caucasian	multivariate	144	71.8	9	5	Baseline BSI	6
Koichi Uemura	2018	Asian	multivariate	48	71.2	10	1	Baseline BSI	6
Suguru Kadomoto	2019	Asian	multivariate	31	70	29	1.797	Baseline BSI, ΔBSI	7
Yasuhide Miyoshi	2019	Asian	multivariate	32	70.7	4	–	ΔBSI	6

### Prognostic value of baseline BSI and ΔBSI for OS in mCRPC patients

**Fig. 2:**
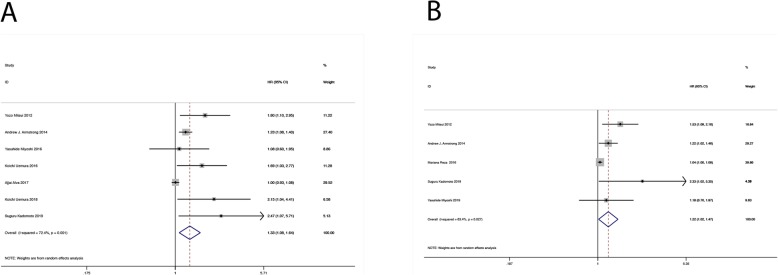
Forest plot HR for the correlation between (**a**) baseline BSI, **b** ΔBSI and OS in mCRPC patients

The association between baseline BSI and ΔBSI for OS in mCRPC patients was estimated by pooled HRs, and 95% CIs are shown in Table 2. All HR data were derived from the results of multivariate analysis, and the results showed that high baseline BSI predicted poor OS (HR = 1.331, 95% CI: 1.081–1.640, *P* = 0.007, Fig. 2a) and that elevated ΔBSI also predicted poor OS (HR = 1.220, 95% CI: 1.015–1.467, *P* = 0.007, Fig. 2b).

**Table 2:** Meta-analysis of baseline BSI, ΔBSI and subgroup for OS

Stratified analysis	Subgroup	No. of studies	P (heterogeneity)	I^2^ (%)	Effect model	HR	(95% CI)	*P*-value	Begg’s test
Baseline BSI	Overall	7	0.001	72.4	Random	1.331	1.081–1.640	0.007	0.764
Ethnicity	Caucasian	2	0.009	85.3	Random	1.102	0.906–1.339	0.331	
	Asian	7	0.485	0	Random	1.688	1.297–2.197	< 0.001	
Cut-off	≤ 1	6	0.274	22.8	Random	1.33	1.072–1.650	0.009	
	> 1	3	0.009	78.9	Random	1.489	0.852–2.604	0.162	
ΔBSI	Overall	5	0.027	63.4	Random	1.22	1.015–1.467	0.034	0.462
Ethnicity	Caucasian	2	0.097	63.7	Random	1.099	0.950–1.272	0.204	
	Asian	3	0.377	0	Random	1.49	1.137–1.954	0.004

### Subgroup analysis

**Fig. 3:**
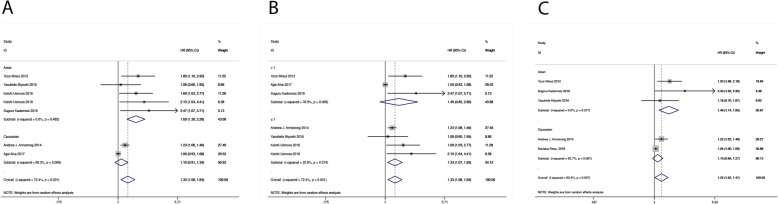
The baseline BSI predicted poor OS in Asian population, in subgroup analyses stratified by (**a**) ethnicity and (**b**) cut-off value. The ΔBSI predicted poor OS in Asian population, in subgroup analyses stratified by (**c**) ethnicity

In subgroup analysis stratified by ethnicity, the baseline BSI predicted poor OS in the Asian population (HR = 1.688, 95% CI: 1.297–2.197, *P* < 0.001, Fig. 3a) but not in the Caucasian population (HR = 1.102, 95% CI: 0.906–1.339, *P* = 0.331, Fig. 3a). In subgroup analysis stratified by cut-off value, the baseline BSI predicted poor OS in the ≤1 population (HR = 1.330, 95% CI: 1.072–1.650, *P* = 0.009, Fig. 3b) but not in the > 1 population (HR = 1.489, 95% CI: 0.852–2.604, *P* = 0.162, Fig. 3b). In subgroup analysis stratified by ethnicity, the ΔBSI predicted poor OS in the Asian population (HR = 1.49, 95% CI: 1.137–1.954, *P* = 0.004, Fig. 3c) but not in the Caucasian population (HR = 1.099, 95% CI: 0.950–1.272, *P* = 0.204, Fig. 3c).

### Publication bias

**Fig. 4:**
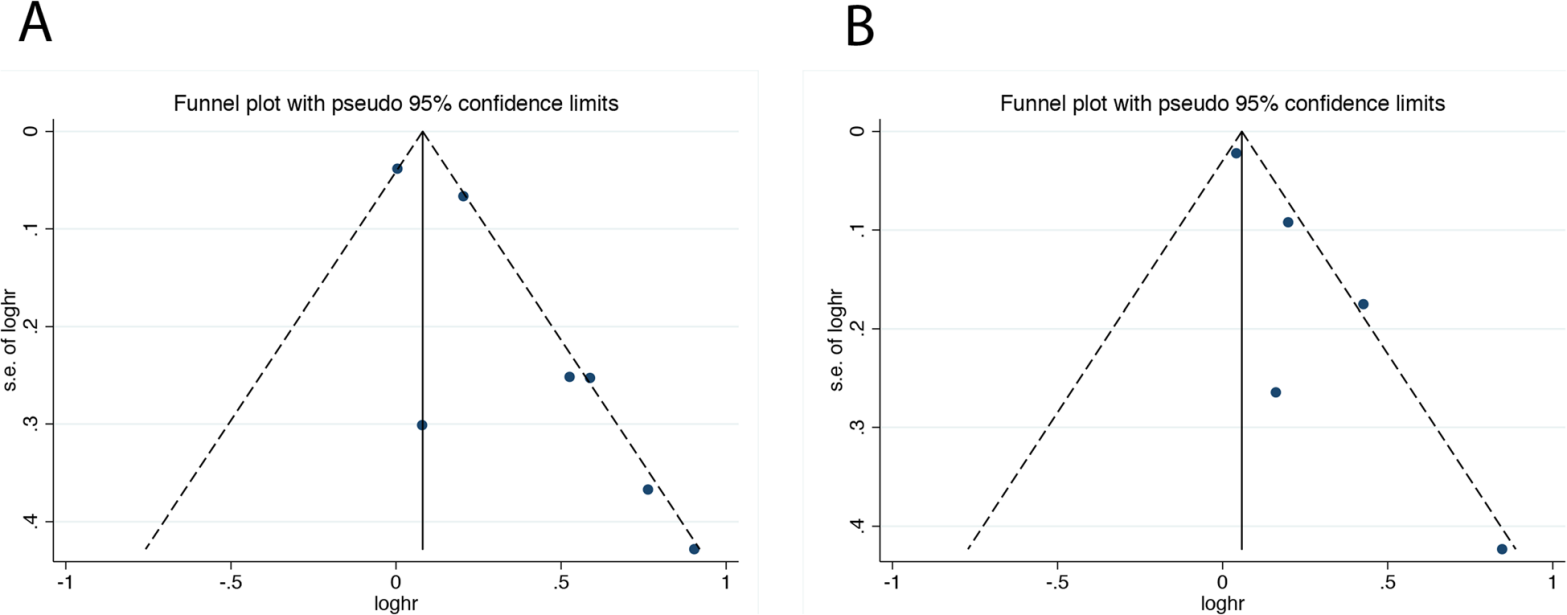
Funnel plot of meta-analysis for (**a**) baseline BSI and (**b**) ΔBSI

Funnel plots of the meta-analysis of baseline BSI (Fig. 4a) and ΔBSI (Fig. 4b) for OS were evaluated for publication bias. Begg’s test evaluated the potential publication bias and is shown in Table 2. The funnel plots and Begg’s test for OS indicated no obvious publication bias.

### Sensitivity analysis

**Fig. 5:**
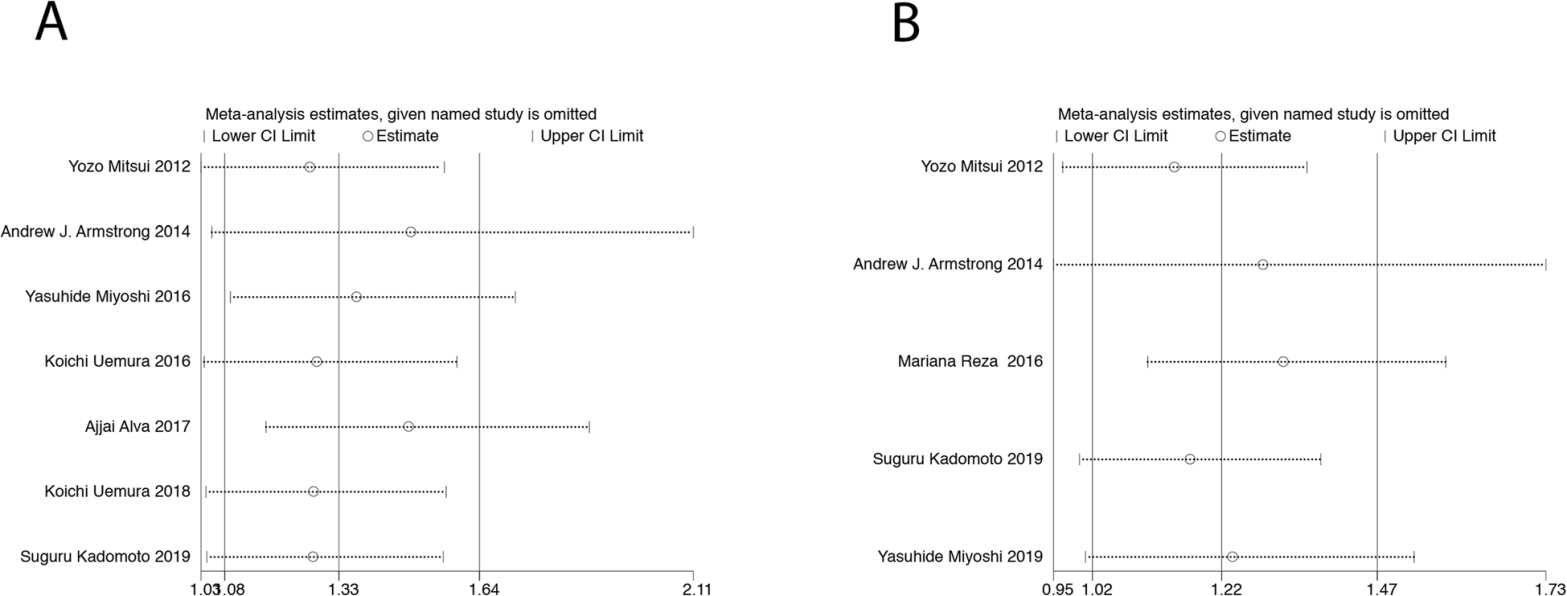
Sensitivity analysis for (**a**) baseline BSI and (**b**) ΔBSI in this meta-analysis

A sensitivity analysis was performed to evaluate the stability of the results and to reduce the effect of the individual studies on the final conclusions. The test suggested that the pooled result of OS for baseline BSI (Fig. 5a) and ΔBSI (Fig. 5b) did not tend to change when an individual study was excluded.

## Discussion

Because more than 80% of patients with mCRPC develop bone metastases [25, 26], the accurate evaluation of these patients is important in assessing prognosis. However, PSA, which is commonly used in clinical practice, is not a good predictor of the clinical prognosis of mCRPC [6, 7]. Therefore, some molecular markers with higher sensitivity and specificity require further discussion.

Many studies have recently shown that BSI progression or a reduction in BSI during treatment was strongly associated with worse OS in men with mCRPC [9, 12, 13, 27]. Among patients receiving taxane chemotherapy, patients with a BSI < 3% had a longer survival time than patients with mCRPC with a BSI ≥3% [21]. Among patients receiving docetaxel for mCRPC, patients with a BSI ≤ 1% survived longer than patients with a BSI greater than 1% [19]. We performed this meta-analysis because the results from the various studies were inconsistent.

We report a systematic review of 567 patients included in 9 studies. The results showed that a high baseline BSI and elevated ΔBSI were significantly associated with poor OS among patients with mCRPC. Li et al. conducted a meta-analysis [28] to research the correlation between the baseline BSI and metastatic prostate cancer (mPCa) prognosis, but the results only showed that the baseline BSI was not associated with OS among patients in a subgroup of mCRPC. Our results are not consistent with those of the aforementioned study. Unfortunately, the researchers who conducted the aforementioned study did not analyse the association between ΔBSI and OS in mCRPC patients. However, regarding ΔBSI, this study systematically estimated the relationship between ΔBSI and the OS of patients with mCRPC.

Population grouping analysis is a serious problem, and it may lead to the evidence related to diseases not being very reliable, suggesting that the environment and the different races have different impacts [29]. In our study, the subgroup analysis stratified by ethnicity showed that the baseline BSI and ΔBSI predicted poor OS in the Asian population but not in the Caucasian population. This is a noteworthy result, which may indicate that different races are not the same in terms of BSI performance. Different studies have used slightly different cut-offs for baseline BSI, which may affect our final results. To further explore the impact of the cut-off value, we performed a subgroup analysis based on the different cut-off values of baseline BSI. The subgroup of ≤1 showed a significant association with OS in mCRPC patients. This suggests that the cut-off value we should choose in future research in this area is less than or equal to 1, rather than higher cut-off values.

We should and must acknowledge that there are some limitations in this study. First, the cut-off criteria to determine the positive or negative baseline BSI were inconsistent in different studies, which may potentially contribute to heterogeneity. Therefore, a more unified standard should be defined in the future, and we suggest that the cut-off value should be less than or equal to 1. Second, the number of people included in this study is limited. Therefore, large-scale multicentre studies are needed to obtain more accurate results. Finally, studies with positive results are more likely to be published than studies with negative results, which may lead to publication bias, although no such bias was found in this analysis [30].

## Conclusions

Our meta-analysis suggests that a high baseline BSI and elevated ΔBSI predicted poor OS among patients with mCRPC. Hence, the BSI can serve as a prognostic indicator for mCRPC patients and may therefore guide clinical treatment in the future. More large-scale clinical trials should be performed to further validate this conclusion.

## Data Availability

The datasets used and analyzed in the present study are available from the corresponding author upon reasonable request.

## Abbreviations

BSI: Bone scan index
mCRPC: metastatic castration resistant prostate cancer
HR: Hazard ratios
CI: Confidence interval
NOS: Newcastle-Ottawa Scale
OS: Overall survival
mPCa: metastatic prostate cancer

## References

[1] Joniau S, Briganti A, Gontero P, Gandaglia G, Tosco L, Fieuws S, Tombal B, Marchioro G, Walz J, Kneitz B, Bader P, Frohneberg D, Tizzani A, Graefen M, van Cangh P, Karnes RJ, Montorsi F, Van Poppel H, Spahn M (2015). Stratification of high-risk prostate cancer into prognostic categories: a European multi-institutional study. Eur Urol.

[2] Small EJ, Vogelzang NJ (1997). Second-line hormonal therapy for advanced prostate cancer: a shifting paradigm. J Clin Oncol.

[3] Oh WK, Kantoff PW (1998). Management of hormone refractory prostate cancer: current standards and future prospects. J Urol.

[4] Sartor O, de Bono JS (2018). Metastatic prostate Cancer. N Engl J Med.

[5] Agarwal N, Di Lorenzo G, Sonpavde G, Bellmunt J (2014). New agents for prostate cancer. Ann Oncol.

[6] Scher HI, Morris MJ, Basch E, Heller G (2011). End points and outcomes in castration-resistant prostate cancer: from clinical trials to clinical practice. J Clin Oncol.

[7] Izumi K, Lin WJ, Miyamoto H, Huang CK, Maolake A, Kitagawa Y, Kadono Y, Konaka H, Mizokami A, Namiki M (2014). Outcomes and predictive factors of prostate cancer patients with extremely high prostate-specific antigen level. J Cancer Res Clin Oncol.

[8] Imbriaco M, Larson SM, Yeung HW, Mawlawi OR, Erdi Y, Venkatraman ES, Scher HI (1998). A new parameter for measuring metastatic bone involvement by prostate cancer: the bone scan index. Clin Cancer Res.

[9] Dennis ER, Jia X, Mezheritskiy IS, Stephenson RD, Schoder H, Fox JJ, Heller G, Scher HI, Larson SM, Morris MJ (2012). Bone scan index: a quantitative treatment response biomarker for castration-resistant metastatic prostate cancer. J Clin Oncol.

[10] Wakabayashi H, Nakajima K, Mizokami A, Namiki M, Inaki A, Taki J, Kinuya S (2013). Bone scintigraphy as a new imaging biomarker: the relationship between bone scan index and bone metabolic markers in prostate cancer patients with bone metastases. Ann Nucl Med.

[11] Ulmert D, Kaboteh R, Fox JJ, Savage C, Evans MJ, Lilja H, Abrahamsson PA, Bjork T, Gerdtsson A, Bjartell A, Gjertsson P, Hoglund P, Lomsky M, Ohlsson M, Richter J, Sadik M, Morris MJ, Scher HI, Sjostrand K, Yu A, Suurkula M, Edenbrandt L, Larson SM (2012). A novel automated platform for quantifying the extent of skeletal tumour involvement in prostate cancer patients using the bone scan index. Eur Urol.

[12] Anand A, Morris MJ, Larson SM, Minarik D, Josefsson A, Helgstrand JT, Oturai PS, Edenbrandt L, Roder MA, Bjartell A (2016). Automated bone scan index as a quantitative imaging biomarker in metastatic castration-resistant prostate cancer patients being treated with enzalutamide. EJNMMI Res.

[13] Reza M, Jones R, Aspegren J, Massard C, Mattila L, Mustonen M, Wollmer P, Tragardh E, Bondesson E, Edenbrandt L, Fizazi K, Bjartell A (2016). Bone scan index and progression-free survival data for progressive metastatic castration-resistant prostate Cancer patients who received ODM-201 in the ARADES multicentre study. Eur Urol Focus.

[14] Liberati A, Altman DG, Tetzlaff J, Mulrow C, Gotzsche PC, Ioannidis JP, Clarke M, Devereaux PJ, Kleijnen J, Moher D (2009). The PRISMA statement for reporting systematic reviews and meta-analyses of studies that evaluate health care interventions: explanation and elaboration. PLoS Med.

[15] Stang A (2010). Critical evaluation of the Newcastle-Ottawa scale for the assessment of the quality of nonrandomized studies in meta-analyses. Eur J Epidemiol.

[16] Miyoshi Y, Uemura K, Kawahara T, Yoneyama S, Hattori Y, Teranishi JI, Ohta JI, Takebayashi S, Yokomizo Y, Hayashi N, Yao M, Uemura H (2017). Prognostic value of automated bone scan index in men with metastatic castration-resistant prostate Cancer treated with Enzalutamide or Abiraterone acetate. Clin Genitourin Cancer.

[17] Reza M, Ohlsson M, Kaboteh R, Anand A, Franck-Lissbrant I, Damber JE, Widmark A, Thellenberg-Karlsson C, Budaus L, Steuber T, Eichenauer T, Wollmer P, Edenbrandt L, Tragardh E, Bjartell A (2016). Bone scan index as an imaging biomarker in metastatic castration-resistant prostate Cancer: a multicentre study based on patients treated with Abiraterone acetate (Zytiga) in clinical practice. Eur Urol Focus.

[18] Kadomoto S, Yaegashi H, Nakashima K, Iijima M, Kawaguchi S, Nohara T, Shigehara K, Izumi K, Kadono Y, Nakajima K, Mizokami A (2019). Quantification of bone metastasis of castration-resistant prostate Cancer after Enzalutamide and Abiraterone acetate using bone scan index on bone Scintigraphy. Anticancer Res.

[19] Uemura K, Miyoshi Y, Kawahara T, Yoneyama S, Hattori Y, Teranishi J, Kondo K, Moriyama M, Takebayashi S, Yokomizo Y, Yao M, Uemura H, Noguchi K (2016). Prognostic value of a computer-aided diagnosis system involving bone scans among men treated with docetaxel for metastatic castration-resistant prostate cancer. BMC Cancer.

[20] Uemura K, Miyoshi Y, Kawahara T, Ryosuke J, Yamashita D, Yoneyama S, Yokomizo Y, Kobayashi K, Kishida T, Yao M, Uemura H (2018). Prognostic value of an automated bone scan index for men with metastatic castration-resistant prostate cancer treated with cabazitaxel. BMC Cancer.

[21] Mitsui Y, Shiina H, Yamamoto Y, Haramoto M, Arichi N, Yasumoto H, Kitagaki H, Igawa M (2012). Prediction of survival benefit using an automated bone scan index in patients with castration-resistant prostate cancer. BJU Int.

[22] Armstrong AJ, Kaboteh R, Carducci MA, Damber JE, Stadler WM, Hansen M, Edenbrandt L, Forsberg G, Nordle O, Pili R, Morris MJ (2014). Assessment of the bone scan index in a randomized placebo-controlled trial of tasquinimod in men with metastatic castration-resistant prostate cancer (mCRPC). Urol Oncol.

[23] Miyoshi Y, Sakamoto S, Kawahara T, Uemura K, Yokomizo Y, Uemura H. Correlation between automated bone scan index change after Cabazitaxel and survival among men with castration-resistant prostate Cancer. Urol Int. 2019;103(3):279–84. 10.1159/000502655 31461725

[24] Alva A, Nordquist L, Daignault S, George S, Ramos J, Albany C, Isharwal S, McDonald M, Campbell G, Danchaivijitr P, Yentz S, Anand A, Yu EY (2017). Clinical correlates of benefit from Radium-223 therapy in metastatic castration resistant prostate Cancer. Prostate.

[25] Petrylak DP, Tangen CM, Hussain MH, Lara PN, Jones JA, Taplin ME, Burch PA, Berry D, Moinpour C, Kohli M, Benson MC, Small EJ, Raghavan D, Crawford ED (2004). Docetaxel and estramustine compared with mitoxantrone and prednisone for advanced refractory prostate cancer. N Engl J Med.

[26] Tannock IF, de Wit R, Berry WR, Horti J, Pluzanska A, Chi KN, Oudard S, Theodore C, James ND, Turesson I, Rosenthal MA, Eisenberger MA (2004). Docetaxel plus prednisone or mitoxantrone plus prednisone for advanced prostate cancer. N Engl J Med.

[27] Mota JM, Armstrong AJ, Larson SM, Fox JJ, Morris MJ (2019). Measuring the unmeasurable: automated bone scan index as a quantitative endpoint in prostate cancer clinical trials. Prostate Cancer Prostatic Dis.

[28] Li D, Lv H, Hao X, Dong Y, Dai H, Song Y (2017). Prognostic value of bone scan index as an imaging biomarker in metastatic prostate cancer: a meta-analysis. Oncotarget.

[29] Hirschhorn JN, Lohmueller K, Byrne E, Hirschhorn K (2002). A comprehensive review of genetic association studies. Genet Med.

[30] Sutton AJ, Song F, Gilbody SM, Abrams KR (2000). Modelling publication bias in meta-analysis: a review. Stat Methods Med Res.

